# Hydrogels as Extracellular Matrix Analogs

**DOI:** 10.3390/gels2030020

**Published:** 2016-08-03

**Authors:** Eva C. González-Díaz, Shyni Varghese

**Affiliations:** Department of Bioengineering, University of California San Diego, 9500 Gilman Drive, La Jolla, CA 92093, USA; ecgonzal@eng.ucsd.edu

**Keywords:** hydrogels, extracellular matrix, bioactive materials

## Abstract

The extracellular matrix (ECM) is the non-cellular component of tissue that provides physical scaffolding to cells. Emerging studies have shown that beyond structural support, the ECM provides tissue-specific biochemical and biophysical cues that are required for tissue morphogenesis and homeostasis. Hydrogel-based platforms have played a key role in advancing our knowledge of the role of ECM in regulating various cellular functions. Synthetic hydrogels allow for tunable biofunctionality, as their material properties can be tailored to mimic those of native tissues. This review discusses current advances in the design of hydrogels with defined physical and chemical properties. We also highlight research findings that demonstrate the impact of matrix properties on directing stem cell fate, such as self-renewal and differentiation. Recent and future efforts towards understanding cell-material interactions will not only advance our basic understanding, but will also help design tissue-specific matrices and delivery systems to transplant stem cells and control their response in vivo.

## 1. Introduction

Reciprocal interactions of cells with their microenvironment are fundamental to multiple cellular processes that are necessary for tissue development, homeostasis, and regeneration [1]. The key players of the microenvironment are the extracellular matrix, cytokines, and growth factors, as well as neighboring cells. The extracellular matrix is a dynamic ensemble of proteins and proteoglycans, which surround cells, provide anchoring sites, and regulate growth factor signaling [2,3,4,5]. While the effect of soluble molecules and growth factors of the microenvironment on cell fate and function is very well understood, our knowledge of the impact of the physicochemical properties of the extracellular matrix is only just beginning to emerge. Emerging evidence has established that the ECM is not just a passive structural support, as previously thought, but is rather an active modulator of various cellular behaviors contributing to tissue morphogenesis and regeneration [1]. The physical and chemical properties of the ECM are tissue-specific and, when negatively perturbed, could contribute to disease progression [6,7]. Biomaterials have played a key role towards our current understanding of the contribution of matrix properties on the cell response. Among different forms of biomaterials, hydrogels have been widely used as three-dimensional (3D) structural supports to culture cells as they provide a highly-hydrated, cytocompatible environment and facilitate nutrient and waste transport [8].

Hydrogels are three-dimensional networks of hydrophilic polymer chains that can imbibe large quantities of biological fluid. Thus, hydrogels are very similar in structure to the ECM of mammalian tissues, which essentially consists of hydrated proteins and polysaccharide networks. Crosslinking of polymeric chains to form hydrogels can be achieved through covalent or non-covalent interactions [8]. Hydrogels have been used to support both monolayer (2D) and 3D cell culture. While most of the initial studies focused on the ability of hydrogels to provide structural support to cells, recent efforts have focused on recapitulating various physicochemical cues of the native ECM. Hydrogels can be created from biologically-derived, naturally occurring, or synthetic precursors [8]. Hydrogels made from biological precursors, such as collagen, possess biochemical cues relevant to various cellular functions such as attachment, growth, and migration. On the other hand, hydrogels made from synthetic precursors are often biologically inert and lack bioactive moieties necessary for supporting cell adhesion. Modification of synthetic hydrogels with bioactive molecules like proteins or peptides is needed to elicit cell adhesion [9,10]. One of the advantages of hydrogels made from synthetic precursors over their biologically-derived counterparts is that their physical properties (e.g., mechanical and degradation properties) can be easily controlled and tuned. The fact that the physicochemical and biological properties of synthetic hydrogels can be easily altered in a reproducible manner makes them ideal candidates to study the effect of ECM properties on cellular functions in a systematic manner.

**Figure 1:**
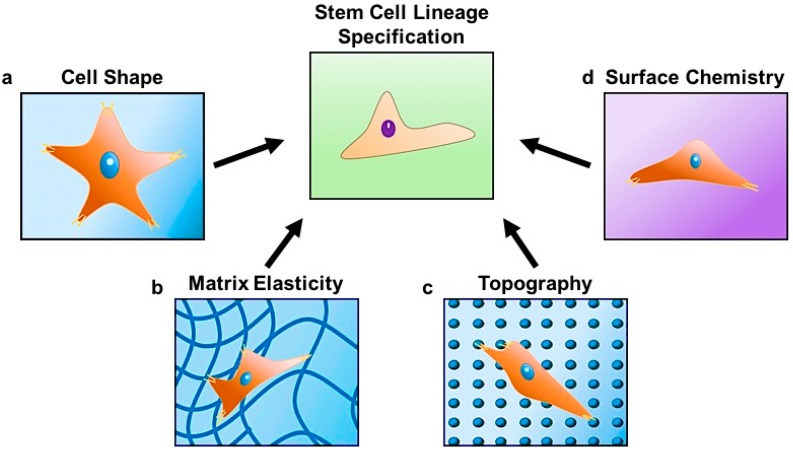
Stem cell lineage specification is regulated by changes in (**a**) cell shape dictated by the surrounding matrix; (**b**) matrix elasticity; (**c**) topography; and (**d**) chemical composition at the cell-material interface.

This review discusses the design of hydrogels with defined physical, chemical, and tunable spatiotemporal features. The review also summarizes how hydrogels with defined surface and bulk properties can be used to regulate various cell functions, including self-renewal and differentiation of stem cells (Figure 1) as well as tissue formation.

## 2. Designing Hydrogels with Defined Physicochemical Properties

Hydrogel properties can be largely divided into surface and bulk properties. These properties can act in concert or individually to regulate various cell functions. In the following sections, we highlight current strategies in the design of hydrogel matrices with tunable surface and bulk properties and their influence on cell behavior.

### 2.1. Design and Synthesis of Cell-Adhesive Hydrogels through Peptide and Protein Immobilization

Hydrogels made from synthetic hydrophilic polymers generally do not support cell adhesion as they are highly resistant to protein adsorption. In fact, the antifouling properties of hydrophilic polymers have been extensively used to improve the longevity of implants, where the modified surfaces prevent protein adsorption and, thereby, failure of the implants. For example, hydrophilic poly(ethylene glycol) (PEG) coatings have been applied to the surface of poly-urethane arterial shunts to reduce clotting resulting from protein adsorption [11]. While such antifouling surfaces/interfaces are advantageous for improving the function of certain implants, this antifouling property limits the application of hydrogels as cell culture substrates, as the minimum requirement for the survival of anchorage-dependent cells is adhesion to the underlying matrix.

A commonly used approach to make hydrogels cell-adhesive is the incorporation of peptides or proteins into the hydrogel network [10,12,13,14,15,16]. The biofunctionalization of hydrogels can be achieved through bioconjugation, such as coupling between –NHS and amine groups [15], Michael-type addition [13], thiol-acrylate reaction [12], or copolymerization [16]. In addition to being inexpensive and easy to synthesize, peptide-immobilized biomaterials are more amenable to sterilization, unlike proteins that could undergo denaturation at non-physiological conditions. The biological activity of immobilized peptides and proteins relies upon their accessibility, suggesting that the tethered groups must be flexible and experience minimal steric hindrance [13]. The most widely studied cell-adhesive peptide is arginine-glycine-aspartate (or RGD), a tri-amino acid sequence. RGD is the key integrin-binding domain present among different ECM proteins [9].

While RGD sequences can assist attachment of cells to biologically inert hydrogels by engaging cell surface integrins, immobilization of RGDs is often not sufficient to regulate biological and signaling events relevant to maintaining self-renewal or directing differentiation of stem cells. This is not surprising, given that the native ECM presents different bioactive units with varying conformation and densities to modulate cellular functions. A number of studies have incorporated peptide units and different ECM components into hydrogels to achieve targeted cellular functions. For instance, Musah et al., endowed poly(acylamide) (PAm) hydrogels with vitronetin-derived peptide units (GKKQRFRHRNRKG) to interact with cell surface glycans [10]. These modified PAm hydrogels displaying glycosaminoglycan binding peptides were found to support self-renewal of human embryonic stem cells (hESCs) in 2D culture [14].

Similar to proteins, polysaccharides of the native ECM also play an important role in mediating cell-matrix interactions. Native ECM proteoglycans are known to regulate growth factor signaling through sequestration and release of growth factors upon cellular demand [5]. Hence, hydrogels have also been designed to regulate growth factor signaling relevant to various cellular functions [17,18,19]. This can be achieved by either functionalizing synthetic hydrogels with proteoglycan moieties [20] or synthetic molecules that mimic proteoglycan functions [17,21,22]. For instance, heparin-functionalized PEG hydrogels have been used to direct osteogenic differentiation of human mesenchymal stem cells (hMSCs) in 3D culture [23]. Heparin is known to sequester growth factors and proteins, such as BMP-2 and fibronectin. Similarly, studies have used hydrogel mineralization to sequester growth factors, such as BMP-2 [24]. The incorporation of chondroitin sulfate (CS) and hyaluronic acid moieties into PEG hydrogels has also been used to impart bioactivity [20,25,26]. A study by Varghese et al. demonstrated that hMSCs encapsulated within CS/PEG hydrogels promoted the formation of cell aggregates and enhanced chondrogenic differentiation and deposition of cartilage-specific ECM [27]. Hydrogels containing hyaluronic acid moieties have been shown to promote chondrogenic differentiation of stem cells, such as MSCs, in 3D cultures [28]. In addition to being a key component of cartilage ECM, hyaluronic acid molecules may interact with encapsulated MSCs through CD44 receptors [29]. Aside from their use as scaffolds for cartilage tissue engineering, hyaluronic acid hydrogels have been explored for a variety of other biomedical applications [30].

While active conjugation of peptides and ECM components can be used to impart bioactivity, non-specific adsorption of proteins can also make hydrogels adhesive to assist cell attachment. A number of parameters, such as surface roughness, chemistry, and hydrophobicity (or hydrophilicity), influence the ability of hydrogels to adsorb proteins [11].

### 2.2. Tuning the Cell-Matrix Interface through Functional Groups and Hydrophobicity

Hydrogels displaying certain functional groups and hydrophobicity can support cell culture in the absence of active immobilization of peptides or proteins, often through non-specific protein adsorption [31]. In general, hydrophobic surfaces have a higher tendency for protein adsorption than do hydrophilic surfaces [11,32]. The adsorbed proteins at the cell-material interface provide cell adhesive domains that support cell attachment and growth. Surface chemistry and hydrophobicity not only influence protein adsorption, but also modulate protein conformation, which in turn regulates integrin binding and cell function [31,33]. In addition to supporting survival and growth, these parameters also play a key role in cell migration and differentiation of stem cells [33,34].

**Figure 2:**
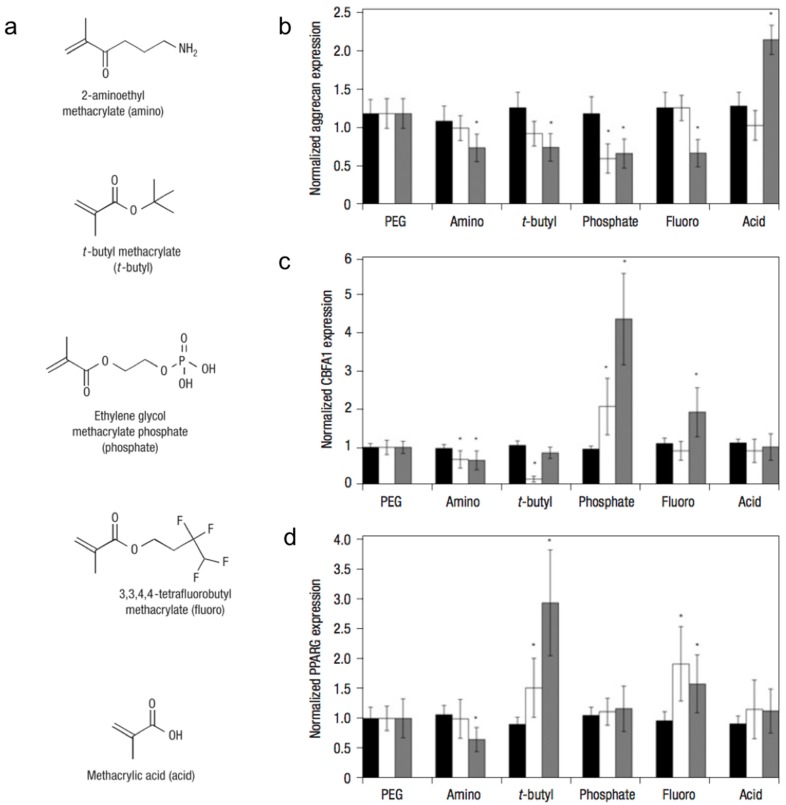
Small-molecule incorporation alters human mesenchymal stem cell (hMSC) gene expression on poly(ethylene glycol) (PEG) hydrogels. (**a**) Chemical structures of functional moieties incorporated. Gene expression of hMSCs (as measured by in situ hybridization) quantitatively analyzed for aggrecan (**b**); CBFA1 (**c**); and PPARG (**d**) at days 0 (black bars), 4 (white bars) and 10 (grey) of culture on unmodified PEG and 50 mM of amino, *t*-butyl, phosphate, fluoro, and acid. Values are reported as the fluorescent intensity average of six samples per composition per time point, relative to β-actin expression, and normalized to expression by cells cultured on PEG surfaces. Error bars represent one standard deviation. An asterisk (*) denotes statistical significance compared with PEG (*p* < 0.05). Adapted with permission from [35]. Copyright 2008 Nature.

By incorporating small-molecules into PEG hydrogel matrices, Benoit et al. examined the effect of matrix functional groups on hMSC differentiation (Figure 2) in 3D culture [35]. Hydrogels containing phosphate functional groups induced osteogenic differentiation, whereas those containing *t*-butyl groups promoted adipogenic differentiation. Additionally, gels that were functionalized with methacrylic acid stimulated the upregulation of cartilage-specific markers, ultimately leading to chondrogenic differentiation. The molecular mechanism by which chemical functional groups induce differentiation of stem cells into a particular phenotype remains unknown.

Hydrogel functional groups have also been used to generate synthetic matrices with bone-specific biochemical cues (i.e., mineral components) [36,37]. In a recent study, we used hydrogels with carboxyl functional groups to generate matrices bearing calcium phosphate (CaP) minerals [38]. These biomineralized hydrogels were found to direct osteogenic commitment of stem cells, such as hMSCs, hESCs, and human induced pluripotent stem cells (hiPSCs), in 2D and 3D cultures, in the absence of any other osteogenic molecules [39,40,41]. The dynamic dissolution (into Ca^2+^ and PO_4_^3−^ ions) and re-precipitation of matrix-bound CaP minerals has been touted to play a key role in the osteoinductivity of these mineralized matrices. This dynamic dissolution and re-precipitation of CaP minerals not only modulates Ca^2+^ and PO_4_^3−^ signaling to influence osteogenic differentiation [42,43,44], but can also sequester and release osteoinductive growth factors such as bone morphogenic proteins (BMPs) [24]. In addition, the CaP minerals of the matrix could contribute to osteogenic commitment of stem cells through PO_4_^3−^-ATP-Adenosine-A2b receptor axis signaling [42] while inhibiting their differentiation into adipogenic lineage [43].

A study by Phillips et al. sought to understand the effect of functional groups on hMSC differentiation by using self-assembled monolayer (SAM) surfaces [34]. Four functional groups: CH_3_, OH, COOH, and NH_2_ were used to represent hydrophobic, hydrophilic, negatively charged, and positively charged interfacial properties, respectively. In this study, surfaces functionalized with –OH and –NH_2_ demonstrated a strong upregulation of osteogenic markers along with a downregulation of adipogenic markers, while showing no significant effect on chondrogenic differentiation. Valamehr et al. used SAM surfaces to examine the effect of substrate hydrophobicity on differentiation of embryonic stem cell-derived embryoid bodies [45]. In another study, hydrogels with low wettability (hydrophobic surfaces) were shown to support clonal growth of hESCs and hiPSCs in 2D culture, through non-specific adsorption of vitronectin [46]. The vitronectin adsorbed onto the surfaces engaged with the cells through *α*_v_*β*_3_ and *α*_v_*β*_5_ integrins and assisted their growth while maintaining pluripotency. A study by Chang et al. incorporated styrene sulfonate functional groups, a potential synthetic analog of heparin, to regulate bFGF signaling and generate hydrogels that could support human pluripotent stem cell growth in monolayer culture and maintain pluripotency ex vivo [21].

While the aforementioned studies demonstrate the influence of matrix interfacial properties on determining various cellular outcomes, it is often difficult to decouple the effect of functional groups and hydrophobicity. A recent study by Ayala et al. addressed this issue by using acryloyl amino acid (AA) monomers with varying side chain lengths (through the number of –CH_2_ groups that separate the vinyl group and terminal –COOH group) [33]. Acrylamide hydrogels functionalized with AA units of varying chain length showed different levels of hydrophobicity without altering the stiffness or the hydrogel functional group. The results from this study showed cells adhered to hydrogels exhibiting an optimal hydrophobicity which grew to confluence, where the adhesion of cells to the underlying matrix was mediated through nonspecific protein adsorption. Based on the shape and alignment of the adhered cells, the cultured hMSCs underwent either osteogenic or adipogenic differentiation.

The hydrophobicity-mediated “adhesivity” of hydrogels has been used to develop “smart surfaces” for cell culture [47,48]. Hydrogels displaying smart surfaces are generally fabricated from polymers exhibiting lower critical solution temperature (LCST). Thus far, poly(*N*-isopropylacrylamide) (pNIPAm) is the most widely-used, temperature-responsive polymer for cell culture. Thermoresponsive hydrogels oscillate between hydrophilic and hydrophobic surfaces around the LCST temperature [49]. At 37 °C, the hydrogel surface is hydrophobic, enabling nonspecific protein adsorption and making the surface cell adhesive. At temperatures below 37 °C, the hydrogel surface becomes hydrophilic and releases the monolayer of cells as a sheet. Such engineered cell sheets have been used to treat a myriad of disorders. For example, engineered myocardial cells sheets have been developed to treat patients suffering from severe heart failure [50]. Such stimuli-responsive hydrogels have also been used for minimally-invasive cell delivery [51], expansion of pluripotent stem cells [52], and multi-functional scaffolds for cell culture [53].

### 2.3. Design of Hydrogels with Topographical Cues

In the human body, the native environment that cells experience is far from flat. The organization of the extracellular matrix gives rise to complex geometrical features, which play a significant role in various cellular functions. A number of studies have employed micropatterned, cell-adhesive geometrical features of various sizes and shapes to examine the effect of cell shape on growth, polarization, migration, and differentiation of stem cells [54,55,56]. Throughout this section, we discuss the most commonly used techniques to generate micropatterned matrices and highlight key studies that have served to expand our understanding of the cell response to topographical cues in 2D. One common micropatterning technique is photolithography, which consists of polymerizing a material by exposing the hydrogel precursor solution to ultraviolet (UV) light through a photomask displaying the desired pattern [57]. A similar approach has been applied to generate hydrogel patterns within microfluidic chips [58,59]. Soft lithographic techniques, such as microcontact printing, involve the use of a master stamp, often made from polydimethylsiloxane (PDMS), that can transfer adhesive proteins or other molecules (referred to as the ink) onto a substrate, to generate patterned features [60,61]. Another approach is micromolding, where the hydrogel precursor solution is placed over a PDMS stamp containing the negative pattern of the desired geometry and allowed to polymerize (usually by exposure to UV light) [62]. The stamp is subsequently removed and the hydrogel is inverted to reveal the patterned surface. Another technique used to generate matrices with topographical features is electrospinning [63]. In this technique, a charged stream of polymer solution is placed within a syringe and exposed to an electric field. The voltage is increased until the electric force generated overcomes the surface tension at the tip of the needle, resulting in the ejection of a jet of polymer that can then be collected on a rotating or stationary collector in the desired orientation. Studies have also used differential swelling of hydrogels as a tool to create surface wrinkles, which can be used to generate different topographical features [55,64].

Findings from studies using micropatterned matrices have shed light into the remarkable manner with which the cytoskeletal architecture of the cell adapts to the shape provided by the substrate surface, subsequently influencing its migration, growth, and differentiation. Specifically, the size and shape of the patterned domains governs the cell volume and spreading, the organization of cytoskeletal networks and, subsequently, intracellular signaling. Results from these efforts have shown that the commitment of MSCs to either an osteogenic or adipogenic lineage can be regulated by the cell shape [65,66]. For instance, cells cultured on the surface of large islands demonstrated increased adhesion and spreading and eventually underwent osteogenesis. In contrast, cells on smaller islands underwent adipogenesis after adopting a round and unspread morphology [65]. The effect of matrix topographical cues on stem cell commitment was further demonstrated by subsequent studies [66] in which geometrical constraints leading to increased actomyosin contractility directed osteogenic differentiation, while those of low contractility led to adipogenic differentiation. Most of these initial seminal studies utilized PDMS, a crosslinked hydrophobic polymer, as a substrate.

**Figure 3:**
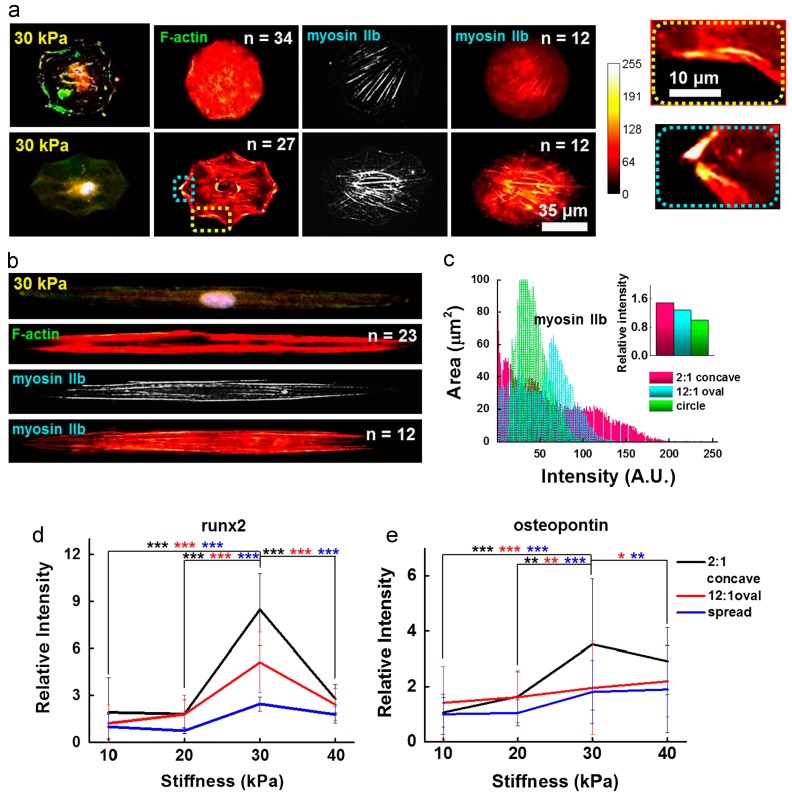
Influence of cell shape on the MSC cytoskeleton. (**a**) and (**b**) show immunofluorescence images and immunofluorescence heatmaps (left to right: F-actin with nuclei, heatmap of F-actin IIb, and heatmap of myosin IIb) for cells cultured in circular, concave, and elongated shape for 10 days; (**c**) heat map intensity comparison for cells stained for myosin IIb. Inset represents myosin IIb intensity normalized to that of circular geometry. Additionally, enhanced osteogenesis marker expression was observed in mesenchymal stem cells patterned in contractile geometries; (**d**) Relative runx2 marker intensity of cells captured on concave or oval shapes or spread on the fibronectin matrix protein, differentiating to osteogenic lineages (*** *p* < 0.0005, *t*-test compared to concave cells on 30 kPa). Runx2 nuclear fluorescence was normalized to cytoplasmic fluorescence. The relative intensity of the fluorescence was determined by comparing each intensity value to the average intensity of spread cells on 10 kPa; (**e**) Relative osteogenic marker intensity (osteopontin) of cells captured on concave or oval shapes or spread on the fibronectin matrix protein (* *p* < 0.05, ** *p* < 0.005, *** *p* < 0.0005, *t*-test compared to concave cells on 30 kPa). The relative intensity of the fluorescence was determined by comparing each intensity value to the average intensity of spread cells on 10 kPa. Adapted with permission from [68]. Copyright 2014 Elsevier.

In recent years, these efforts have been extended towards the creation of hydrogels with topographical features. For instance, Lee et al. studied the effect of geometric confinement on MSC spreading and lineage specification by patterning ECM proteins, such as fibronectin, laminin, and type I collagen, over the surface of hydrazine-treated polyacrylamide hydrogels [67]. Cells that were more spatially constrained adopted a round morphology and ultimately underwent adipogenic differentiation. In contrast, cells that were allowed to spread freely over the hydrogel surface showed upregulation of neurogenic markers. A similar study used micropatterned polyacrylamide hydrogels to demonstrate the effect of 2D geometric cues on osteogenic differentiation of MSCs [68]. In this study, osteogenesis was enhanced when cells where cultured on geometric shapes that generated an increase in cytoskeletal tension, as was observed for cells growing on elongated shapes (Figure 3).

Surface grates, commonly consisting of parallel lines of defined width and depth, have been employed to enhance cell adhesion and guide cell polarization [69,70]. Specifically, this technique has proven successful in directing neurite extension of PC12s, a neuronal progenitor cell line, as these extensions form parallel to the axis of the grates [71]. Additionally, seeding hMSCs over nanogrates that were 350 nm wide resulted in an upregulation of microtubule-associated protein 2 (MAP2), a key marker in neuronal differentiation [72]. Using a similar concept, nanopitted surfaces have been used to study hMSC differentiation. Interestingly, identical pit dimensions can be used for entirely different purposes, ranging from stem cell maintenance [73] to osteogenesis [74], by simply varying their spatial arrangement.

## 3. Design of Hydrogel Bulk Properties to Probe and Direct Cell Function

### 3.1. Tuning Matrix Stiffness to Guide Cell Behavior

The human body is comprised of tissues with vastly different mechanical properties, ranging from soft tissue, such as that found in the brain, to the stiff tissue that constitutes bone. This has led to activities examining the role of matrix mechanical properties on cell and tissue functions. Hydrogels have been extensively used to study the effect of matrix mechanical properties, such as Young’s modulus (commonly termed as stiffness) on cell function both in vitro and in vivo. Hydrogel matrix stiffness can be varied by controlling the network crosslink density. The network crosslink density can be increased by increasing the concentrations of the crosslinker and/or the monomer or oligomer concentration [75]. Other approaches to improve the mechanical properties of hydrogels include the incorporation of hydrophobic domains (to control swelling) [76], nanoclays (which act as physical crosslinks) [77,78], sacrificial chains, or by unzipping ionic crosslinks (such as in the case of double-network hydrogels) [79,80].

**Figure 4:**
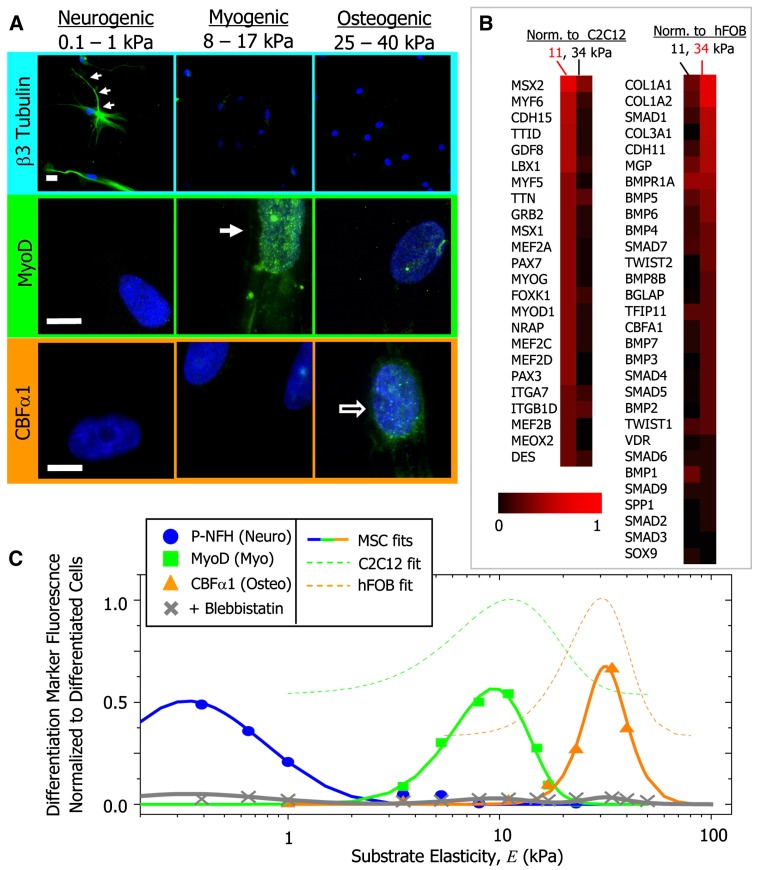
Protein and transcript profiles are elasticity dependent under identical media conditions (**A**) The neuronal cytoskeletal marker b3 tubulin is expressed in branches (arrows) of initially naive MSCs (>75%) and only on the soft, neurogenic matrices. The muscle transcription factor MyoD1 is upregulated and nuclear localized (arrow) only in MSCs on myogenic matrices. The osteoblast transcription factor CBFa1 (arrow) is likewise expressed only on stiff, osteogenic gels. Scale bar is 5 mm; (**B**) Microarray profiles of MSCs cultured on 11 or 34 kPa matrices, with expression normalized first to actin and then to expression of committed C2C12 myoblasts and hFOB osteoblasts; (**C**) Fluorescent intensity of differentiation markers versus substrate elasticity reveals maximal lineage specification at the *E* typical of each tissue type. Average intensity is normalized to peak expression of control cells (C2C12 or hFOB). Adapted with permission from [85]. Copyright 2006 Elsevier.

In vitro studies using hydrogels have demonstrated that matrix mechanical properties play a crucial role in stem cell phenotypic expression by influencing cell shape and mechanotransduction [81]. Essentially, matrix stiffness has been shown to influence various cell functions, including cell adhesion [82,83], proliferation [84], migration [82,83], and stem cell differentiation [85]. For example, proliferation of neural stem cells in 3D hydrogels increases when the elastic modulus is decreased from ~20,000 Pa to ~180 Pa [84]. Stiffer matrices also allow for stronger cell adhesion and decrease the rate of cell migration [86]. Two dimensional studies have shown that when cultured on hydrogels exhibiting a stiffness gradient, cells undergo directed migration towards higher matrix stiffness [82]. A seminal study performed by Engler et al. demonstrated that matrix elasticity can play a key role in directing stem cell differentiation (Figure 4) in 2D culture [85]. The authors showed that preconditioned hMSCs that were cultured on hydrogels with an elastic modulus (E) ranging from 0.1–1 kPa underwent neurogenesis, while those cultured on stiffer hydrogels of modulus ranging from 8–17 kPa and 25–40 kPa underwent myogenesis and osteogenesis, respectively. Similarly, a number of studies have documented the importance of matrix mechanical properties on maintaining cellular functions [83,87,88].The effect of matrix stiffness is also evaluated in 3D culture [89,90]. Studies by Khetan et al. [90] utilizing degradable hydrogels have demonstrated the role of traction stresses generated by encapsulated hMSCs on their fate commitment. Essentially, hydrogel networks that permitted high traction stresses of hMSCs supported their osteogenic differentiation, whereas those with low traction stresses stimulated adipogenic differentiation. Cells respond to matrix rigidity by exerting traction forces on the surrounding matrix through focal adhesions. These integrin binding sites serve as a line of mechanical communication with the cell cytoskeleton, such that increased resistance to deformation in the matrix is reflected by an increase in cytoskeletal tension. Changes in cytoskeletal tension and actomyosin contractility have been shown to trigger various signaling cascades, such as RhoA signaling, that influence transcriptional regulation of associated genes. For instance, an increase in RhoA signaling has been shown to direct osteogenic commitment of MSCs, while a decrease in RhoA promotes adipogenic differentiation [65].

**Figure 5:**
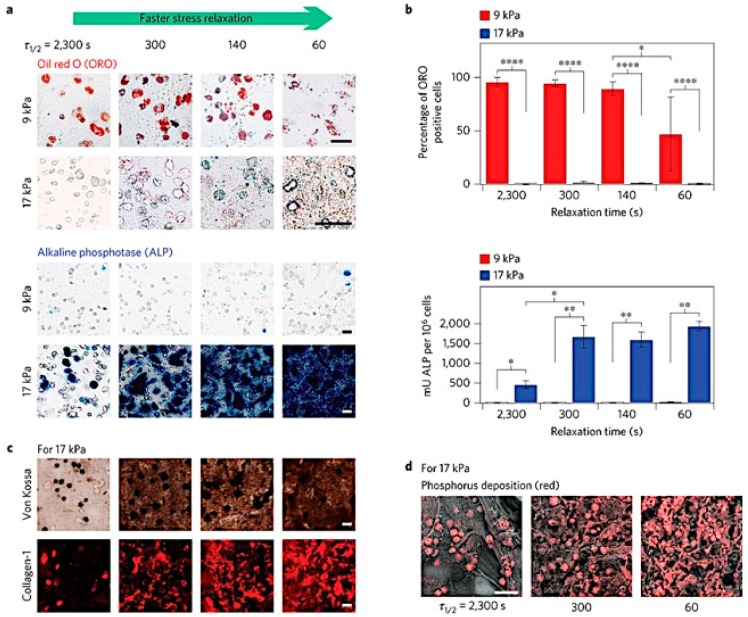
MSCs undergo osteogenic differentiation and form an interconnected mineralized collagen-1-rich matrix only in rapidly relaxing gels. (**a**) Oil Red O staining (red), indicating adipogenic differentiation, and alkaline phosphatase staining (blue), indicating early osteogenic differentiation, for MSC cultured in gels of indicated initial modulus and timescale of stress relaxation for seven days. Scale bars are 25 µm; (**b**) Percentage of cells staining positive for Oil Red O, and a quantitative assay for alkaline phosphatase activity. *, **, and **** indicate *p* < 0.05, 0.01, and 0.0001 respectively (Student’s *t*-test); (**c**) Von Kossa (mineralization) and collagen-1 stain on cryosections from gels with the indicated conditions after two weeks of culture. Scale bars are 25 µm; (**d**) Scanning electron microscope and energy-dispersive X-ray spectrometry (SEM-EDS) images of sections of gels with the indicated conditions after two weeks of 3D culture of MSCs. Phosphorus elemental maps (P mapped in red) are overlaid on their corresponding backscattered SEM images. Scale bar is 50 µm. Adapted with permission from [92]. Copyright 2015 Nature.

In native tissue, the traction forces that cells exert on the surrounding ECM, along with the mechanical properties of the matrix, dictate the extent to which cells are able to remodel their environment. In turn, the resistance to traction forces decreases over time, thus influencing cell behavior [91]. Recently, Chaudhuri et al. created reversible, 3D alginate hydrogels with stress relaxation properties to understand the effect of non-linear mechanical properties of the ECM on cell functions [92]. Hydrogels with a faster rate of stress relaxation not only improved cell spreading [91,92] and proliferation, but also induced osteogenic differentiation of MSCs (Figure 5) [92]. The mechanical properties of the matrix also have a significant effect in local clustering of RGD ligands, actomyosin contractility, as well as the nuclear translocation of YAP (Yes-associated protein), a key transcriptional regulator involved in stem cell differentiation [93,94].

Efforts have also been made to dynamically tune matrix elasticity and recapitulate certain dynamic features of native ECM. To this end, Stowers et al. have created 3D alginate hydrogels (through Ca^2+^ mediated gelation) embedded with light-sensitive liposomes [95]. Encapsulation of either calcium or DTPA (a chelating agent) into these liposomes allowed for the light-triggered and spatially-controlled stiffening or softening of the hydrogel. This design was used to investigate the effect of hydrogel stiffening dynamics on cell morphology using 3T3 fibroblasts. Hydrogels containing encapsulated cells were irradiated for 30, 60, and 120 s, resulting in an increase in stiffness that was proportional to the irradiation time. While fibroblasts that were encapsulated in the unirradiated hydrogel (control group) retained an elongated morphology, cells in the irradiated hydrogels exhibited a round morphology when adapting to the increasing hydrogel stiffness. As a proof of concept, light-triggered stiffening of the hydrogels was also successfully achieved in vivo through transdermal irradiation of the constructs after subcutaneous implantation in mice. In addition, other approaches have been used, such as thiolene polymerization [96] and incorporation of photocleavable moieties [97], to manipulate hydrogel mechanical properties post-encapsulation in culture.

Similarly, a study by Guvendiran and Burdick investigated the effect of dynamic substrate stiffening by using methacrylated hyaluronic acid [98]. Hydrogels were initially crosslinked through Michael-type addition reaction using dithiothreitol (DTT) at various concentrations to tune the initial matrix stiffness (between ~3 and ~100 kPa). The unreacted methacrylate groups were subsequently used to increase the stiffness of the hydrogels through photopolymerization. Within 4 h, both the mean area and the average traction of the encapsulated hMSCs increased in response to an increase in stiffness in the hydrogel network (from ~3 to ~30 kPa). Additionally, the hMSCs differentiated into different phenotypes depending on the amount of time they were cultured in either soft or stiff hydrogels. Adipogenic differentiation was observed with late stiffening of the matrices while osteogenic differentiation was observed in cells cultured in hydrogels with early stiffening.

### 3.2. Designing Pore Architecture to Promote Tissue Formation

Efficient nutrient and waste transport is key to long term cell survival and tissue formation. Hence, matrix porosity plays a significant role in 3D cell culture and engineering of functional tissues from stem cells. Furthermore, matrices with porosity and interconnectivity have also been shown to promote host cell infiltration, homogeneous cell distribution, and integration with the surrounding native tissue. Throughout this section, we highlight recent studies investigating the effect of pore architecture on cell function and tissue formation in 3D.

Porous hydrogels can be generated using a variety of methods, such as solvent casting and particle leaching [99,100], freeze-drying [101], electrospinning [102,103,104], and gas foaming [105]. Pore architecture must be chosen with a tissue-specific context in mind to improve cell function. For instance, Zeng et al. studied the effect of pore size on chondrocyte growth and function using microcavitary alginate hydrogels [106]. Porcine chondrocytes were encapsulated within matrices of various pore size ranges: 80–120 μm, 150–200 μm, and 250–300 μm. After 21 days, cells that were cultured in hydrogels with pore sizes of 80–120 μm exhibited better growth and maintenance of the chondrocyte phenotype.

The pore architecture (pore size, porosity, and pore interconnectivity) of the matrix must also facilitate cell infiltration and angiogenesis when implanted in vivo. Angiogenesis is particularly important for maintaining cell viability and promoting integration of the engineered tissue with the host tissue. The importance of vascularization becomes increasingly apparent in therapeutic strategies that involve cell transplantation, as poor cell survival often limits the potential benefits of the implant. Oliviero et al. developed VEGF-loaded, porous PEG-co-heparin hydrogels to promote angiogenesis and reported that using a pore size range of 35 to 100 μm and a total porosity of ~50.8% is optimal for promoting neovascularization [107]. Similarly, Dziubla et al. investigated the effect of pore size and porosity of poly(2-hydroxyethylmethacrylate) (PHEMA)-based hydrogels on in vitro tubule formation of human microvascular endothelial cells (HMVECs) [108]. Gels were synthesized with pore sizes ranging from ~5 to ~16 μm and with porosities ranging from ~55% to ~90%. While hydrogels with pore sizes lower than ~8 μm showed minimal cell infiltration and vascularization, those with average pore size above ~9 μm and having ~85% porosity or higher exhibited optimal tubule formation and penetration throughout the structures. These tubules were ~7.5–7.85 μm in diameter and had an average tubule length of ~88–102 μm. In another study, Matsiko et al. investigated the effect of matrix pore size on chondrogenic differentiation of MSCs using collagen-hyaluronic acid hydrogels. Their studies showed that matrices with a mean pore size of 300 μm promoted proliferation and chondrogenic differentiation of MSCs when compared to those with smaller mean pore size (94 and 130 μm) [109]. It is important to note that while vascularization is essential for functional engineering of most tissues, matrix architecture should be designed to avoid angiogenesis when dealing with avascular tissues, such as cartilage.

Another key design consideration for implant success is pore interconnectivity. A study by Bakshi et al. used PHEMA hydrogels with interconnected pores of size ranging from 10 to 20 μm and examined their effect on axonal regeneration in vivo. After soaking in brain-derived neurotrophic factor (BDNF), these constructs not only demonstrated significant angiogenesis, but also served as a “bridge” to promote axonal penetration and regeneration after spinal cord injury [110]. Phadke et al. investigated the effect of pore architecture on osteogenic differentiation of hMSCs [111]. Specifically, CaP-mineralized PEGDA-*co*-A6ACA hydrogels were generated with either a randomly-oriented, “spongy” pore architecture (~50–60 μm pore size) or a directional, columnar pore structure (~100–150 μm pore size). hMSCs that grew on spongy cryogels demonstrated a more spread morphology and showed a higher upregulation of osteogenic markers, such as RUNX2, osteopontin, and osteocalcin in vitro.

Aside from pore architecture, the degradation of the matrix also plays a key role in determining tissue formation [112,113]. In an ideal scenario, a scaffold is expected to degrade at a rate that will accommodate cell-secreted ECM without impeding the production of ECM by the cells. A number of different approaches can be adopted to achieve scaffold degradation. This includes incorporation of functional groups, such as poly(esters), that are labile to hydrolytic degradation [114], peptide sequences that are cleavable by proteases, such as matrix metalloproteinases (MMPs) [115], plasmin [116], and elastase [117], and functional moieties that are labile to cell secreted molecules, such as glutathione or thiol-group containing molecules [118].

## 4. Conclusions

Recent fundamental and technological advancements have significantly improved our understanding of the active participation of hydrated ECM on various cellular functions, ranging from survival to phenotypic commitment. Improvements in the formulation of biomimetic hydrogels that incorporate tissue-specific biochemical and biophysical cues to control stem cell lineage specificity in vitro and in vivo have been truly dramatic in recent years. Beyond expanding our basic understanding of stem cell biology, many of these developments have significantly advanced the field of regenerative medicine and its prospect of moving from the bench to the bedside. However, widespread clinical application of these advancements still relies on our ability to standardize the manufacturing and scale-up processes. Nonetheless, there is no doubt that novel methods at the interface of biomaterial manipulation and stem cell biology will continue to be successfully used to propel the advancement of regenerative medicine and translation of stem cell-based therapeutics.
